# The Plantation

**Published:** 1873-08

**Authors:** 


The Plantation is one of the best-looking of our agricultural exchanges. Its contents are always varied and interesting. It numbers among its contributors some of the best agriculturalists of the country. Published at Atlanta, Georgia, by the Plantation Publishing Company, at $1.50 per annum.

